# Impact of early enteral nutrition combined with bedside rehabilitation on functional outcomes and length of stay in patients with severe subarachnoid hemorrhage: a retrospective cohort study

**DOI:** 10.3389/fneur.2026.1768799

**Published:** 2026-04-08

**Authors:** Chunhong Ma

**Affiliations:** Department of Neurology, Nanjing First Hospital, Nanjing Medical University, Nanjing, Jiangsu, China

**Keywords:** bedside rehabilitation, early enteral nutrition, functional outcome, modified Rankin Scale, neurocritical care, subarachnoid hemorrhage

## Abstract

**Objective:**

Early enteral nutrition (EEN) and early rehabilitation have individually demonstrated benefits in neurocritical care. However, the synergistic effects of combining these interventions in severe subarachnoid hemorrhage (SAH) remain unexplored. This study aimed to evaluate the impact of a combined early enteral nutrition and bedside rehabilitation protocol on functional outcomes and length of stay in patients with severe SAH.

**Methods:**

This retrospective cohort study included 102 patients with severe SAH (Hunt–Hess grade III–V) admitted to the neurosurgical intensive care unit (NSICU) between August 2023 and August 2025. Patients were divided into a combined intervention group (*n* = 53), receiving EEN within 48 h and comprehensive bedside rehabilitation within 72 h, and a standard care group (*n* = 49). The primary outcome was favorable functional status [modified Rankin Scale (mRS) 0–2] at 6 months. Secondary outcomes included mRS at discharge and 90 days, length of stay, complications, and mortality.

**Results:**

Favorable outcome at 6 months was significantly higher in the combined intervention group compared to standard care (64.2% vs. 30.6%, *P* = 0.001). Multivariable logistic regression identified combined intervention as an independently associated with favorable outcome (adjusted OR: 3.42, 95% CI: 1.38–8.47, *P* = 0.008). The intervention group demonstrated significantly shorter ICU stay (13.5 vs. 19.8 days, *P* = 0.001) and hospital stay (22.4 vs. 26.9 days, *P* = 0.004). Rates of delayed cerebral ischemia (DCI; 13.2% vs. 42.9%, *P* = 0.002), cerebral vasospasm (15.1% vs. 36.7%, *P* = 0.023), and in-hospital mortality (0% vs. 14.3%, *P* = 0.005) were significantly lower in the intervention group.

**Conclusions:**

Combined early enteral nutrition and bedside rehabilitation was associated with improved functional outcomes and reduced length of stay in severe SAH patients. This multimodal support further investigation of this multimodal approach in prospective trials.

## Introduction

Aneurysmal subarachnoid hemorrhage (SAH) remains a devastating cerebrovascular event with significant morbidity and mortality, affecting approximately 9 per 100,000 individuals annually worldwide (1). Despite advances in neurosurgical techniques and intensive care management, nearly half of SAH survivors experience persistent functional disability, imposing substantial burdens on patients, families, and healthcare systems (2, 3). The pathophysiology of SAH involves complex cascades of early brain injury, neuroinflammation, and delayed cerebral ischemia (DCI), which collectively contribute to poor outcomes (4).

Nutritional support in neurocritical care has garnered increasing attention as a modifiable factor influencing patient outcomes. The hypermetabolic and hypercatabolic state following severe brain injury accelerates protein breakdown and muscle wasting, compounding the risk of ICU-acquired weakness and prolonged mechanical ventilation (5, 6). Current evidence supports early enteral nutrition (EEN) initiation within 24–48 h in critically ill patients, as recommended by both the European Society for Clinical Nutrition and Metabolism (ESPEN) and the American Society for Parenteral and Enteral Nutrition (ASPEN) (7, 8). In SAH specifically, Suzuki et al. demonstrated that high-protein enteral formulas improved functional outcomes at discharge, with an adjusted odds ratio of 2.45 for favorable mRS (9). Similarly, Kofler et al. (10) reported that higher protein delivery was independently associated with better outcomes in SAH patients who developed infectious complications.

Parallel to nutritional optimization, early mobilization and rehabilitation have emerged as critical components of neurocritical care bundles. A landmark propensity-matched study by Takara et al. (11) demonstrated that walking training initiated within 14 days of SAH onset resulted in favorable outcomes (mRS 0–2) in 81.1% vs. 52.5% of patients receiving delayed mobilization. Yang et al. (12) further showed that ICF-based progressive mobilization protocols reduced pneumonia rates and shortened ICU stay without increasing adverse events. These findings align with the growing recognition that prolonged immobilization in the ICU contributes to physical deconditioning, cognitive impairment, and delayed functional recovery (13).

Despite compelling evidence supporting individual interventions, a critical knowledge gap exists regarding the combined effects of early nutrition and early rehabilitation in neurocritical care populations. Zhou et al. (14) demonstrated in the EMAS trial that combined early mobilization and nutrition support reduced ICU-acquired weakness in general ICU patients; however, this trial explicitly excluded patients with neurological conditions. The potential for synergistic benefits—whereby optimal nutritional substrate availability enhances the anabolic response to physical rehabilitation—provides a strong biological rationale for evaluating combined protocols (15).

Therefore, the present study aimed to evaluate the impact of a combined early enteral nutrition and comprehensive bedside rehabilitation protocol on functional outcomes and length of stay in patients with severe SAH. We hypothesized that this multimodal intervention would improve 6-month functional outcomes compared to standard care, with secondary benefits including reduced hospitalization duration and complication rates.

## Materials and methods

### Study design and setting

This retrospective cohort study was conducted at the Neurosurgical Intensive Care Unit (NSICU) of Nanjing First Hospital, a tertiary academic medical center affiliated with Nanjing Medical University. The study protocol was approved by the Institutional Review Board of Nanjing First Hospital (approval number: NJH-2025-089). Due to the retrospective nature of the study, the requirement for individual informed consent was waived. The study adhered to the Strengthening the Reporting of Observational Studies in Epidemiology (STROBE) guidelines (16).

### Patient selection

We screened consecutive patients admitted to the NSICU with spontaneous SAH between August 2023 and August 2025. Inclusion criteria were: (1) age ≥18 years; (2) aneurysmal SAH confirmed by computed tomography angiography or digital subtraction angiography; (3) severe SAH defined as Hunt–Hess grade III–V; (4) aneurysm secured by surgical clipping or endovascular coiling within 72 h of admission; and (5) NSICU stay ≥7 days. Exclusion criteria included: (1) traumatic or non-aneurysmal SAH; (2) contraindications to enteral feeding (active gastrointestinal bleeding, intestinal obstruction, or abdominal compartment syndrome); (3) pre-existing severe disability (baseline mRS >2); (4) pregnancy; (5) terminal malignancy or comfort measures only status; and (6) incomplete medical records or loss to follow-up.

### Group definitions and interventions

Patients were categorized into two groups based on the timing and implementation of nutritional and rehabilitation interventions. The combined intervention group received both EEN initiated within 48 h of aneurysm treatment AND comprehensive bedside rehabilitation initiated within 72 h of aneurysm treatment. The standard care group included patients who received delayed enteral nutrition (>48 h) or inconsistent rehabilitation implementation.

The early enteral nutrition protocol involved nasogastric or nasojejunal tube placement within 24 h of aneurysm treatment, with feeding initiated at 20 ml/h and advanced per tolerance to achieve ≥80% of calculated energy requirements (25 kcal/kg/day) and protein targets (≥1.2 g/kg/day) by day 7. A high-protein peptide-based formula (Peptisorb, Nutricia) was preferentially used (17). Gastric residual volume was monitored every 4 h, with prokinetic agents administered for gastroparesis. Albumin infusion was administered per institutional protocol when serum albumin fell below 25 g/L. Transition to oral diet was initiated following bedside swallowing evaluation and, when indicated, videofluoroscopic swallowing study, with texture modifications based on swallowing function.

The comprehensive bedside rehabilitation protocol encompassed three components: (1) progressive physical therapy including passive range of motion, active-assisted exercises, bed mobility training, sitting balance, standing, and ambulation as tolerated; (2) swallowing rehabilitation with videofluoroscopic evaluation, oro-motor exercises, and modified diet textures; and (3) cognitive stimulation through orientation activities, verbal communication, and simple task engagement (18, 19). Rehabilitation sessions were conducted twice daily by certified physical and occupational therapists. Safety parameters included stable intracranial pressure (< 20 mmHg), mean arterial pressure 70–110 mmHg, absence of active bleeding, and adequate sedation scores (Richmond Agitation-Sedation Scale −2 to +1) (20).

### Data collection

Baseline demographic and clinical characteristics were extracted from electronic medical records, including age, sex, Hunt–Hess grade, World Federation of Neurosurgical Societies (WFNS) grade, modified Fisher scale, admission Glasgow Coma Scale (GCS), Acute Physiology and Chronic Health Evaluation II (APACHE II) score, aneurysm location and treatment modality, and comorbidities. Intervention-related variables included time to enteral nutrition initiation, time to rehabilitation initiation, caloric and protein achievement rates at day 7, and rehabilitation adherence.

Serum albumin and prealbumin levels were recorded at baseline and day 14. Consistent with current ESPEN and ASPEN recommendations, these parameters were analyzed as prognostic inflammatory markers reflecting acute-phase response severity rather than as direct indicators of nutritional status, given their well-documented sensitivity to inflammation and catabolic stress in critically ill patients. Complications were documented according to standardized definitions: DCI was diagnosed per the consensus criteria of Vergouwen et al. (21); cerebral vasospasm was defined by transcranial Doppler mean flow velocity >120 cm/s or angiographic narrowing >50%; pneumonia was diagnosed per CDC/NHSN criteria; and hydrocephalus requiring external ventricular drainage or ventriculoperitoneal shunting was recorded.

### Methodological considerations

Several design features of this retrospective study warrant explicit acknowledgment. First, exposure assignment based on timing thresholds (EEN within 48 h of aneurysm treatment, rehabilitation within 72 h of aneurysm treatment) may introduce time-dependent bias, as patients must survive and remain clinically stable to receive the full intervention protocol. To partially address this concern, we required aneurysm securement within 72 h of admission for all patients, providing a common anchor point for exposure timing. Second, the NSICU stay ≥7 days inclusion criterion was implemented to ensure adequate observation time for intervention implementation and complication ascertainment. We acknowledge this criterion may preferentially exclude early deaths and rapid recoveries, potentially enriching the cohort for intermediate-prognosis patients. This selection effect may limit generalizability to the broader severe SAH population and could influence apparent treatment effects in either direction. Third, the standard care group was defined as patients receiving delayed enteral nutrition (>48 h) or inconsistent rehabilitation, which represents a heterogeneous comparator including varying levels of care intensity. This heterogeneity may affect effect size estimates. Fourth, given the retrospective, non-randomized design, residual confounding by unmeasured variables—such as treating physician preferences, nursing care intensity, or subtle differences in illness trajectory—cannot be excluded despite multivariable adjustment.

### Outcome measures

The primary outcome was favorable functional status at 6 months, defined as modified Rankin Scale (mRS) score 0–2. Assessments at 90 days and 6 months were performed via standardized structured telephone interviews using the simplified modified Rankin Scale questionnaire by two certified stroke nurses who were not involved in acute patient care and were blinded to intervention group assignments. Inter-rater reliability was established through independent scoring of 20 cases (weighted kappa = 0.85). For the discharge assessment, mRS was determined by treating the rehabilitation physician based on documented functional status in the medical record. Six-month mortality, corresponding to mRS 6, is reported explicitly in the results (22). Secondary outcomes included: (1) favorable mRS at discharge and 90 days; (2) ICU and hospital length of stay; (3) mechanical ventilation duration; (4) complication rates (pneumonia, DCI, cerebral vasospasm, hydrocephalus, gastrointestinal intolerance); (5) in-hospital, 90-day, and 6-month cumulative mortality; and (6) biochemical parameters at day 14. Cumulative mortality at each timepoint was defined as death occurring at any point up to and including that assessment, ensuring logical consistency across follow-up periods.

### Statistical analysis

Continuous variables were assessed for normality using the Shapiro–Wilk test. Normally distributed data are presented as mean ± standard deviation (SD) and compared using independent *t*-tests; non-normally distributed data are presented as median [interquartile range (IQR)] and compared using Mann–Whitney *U*-tests. Categorical variables are presented as frequencies (percentages) and compared using chi-square tests or Fisher's exact tests as appropriate.

Multivariable logistic regression was performed to identify factors independently associated with favorable outcome at 6 months, adjusting for clinically relevant covariates selected *a priori*: age, sex, Hunt–Hess grade, admission GCS, APACHE II score, modified Fisher scale, aneurysm treatment modality, hypertension, and admission body mass index (BMI). Continuous variables were assessed for linearity assumption using restricted cubic splines with three knots; no significant departures from linearity were detected. Multicollinearity was evaluated using variance inflation factors (VIF), with all values < 2.5 indicating acceptable collinearity. With 55 favorable outcomes at 6 months and 10 covariates, the events-per-variable ratio was 5.5:1, which we acknowledge approaches the minimum threshold for model stability (23). Results are expressed as adjusted odds ratios (ORs) with 95% confidence intervals (CIs). Model calibration was assessed using the Hosmer–Lemeshow goodness-of-fit test, calibration plot (Supplementary Figure 1), and Brier score. Discrimination was evaluated by the area under the receiver operating characteristic curve (AUC). As a sensitivity analysis to address potential confounding, we constructed a propensity score for receiving combined intervention using all baseline covariates. Inverse probability of treatment weighting (IPTW) was applied to estimate the average treatment effect, with stabilized weights truncated at the 1st and 99th percentiles to reduce the influence of extreme weights.

The primary outcome (mRS 0–2 at 6 months) was prespecified. Secondary outcomes and complication rates were considered exploratory, and *P*-values for these endpoints are presented without adjustment for multiplicity and should be interpreted as hypothesis-generating. A two-sided *P*-value < 0.05 was considered statistically significant. All statistical analyses were performed using Python 3.11 (Python Software Foundation) with SciPy 1.11 and scikit-learn 1.3 packages, and R version 4.3.1 (R Foundation for Statistical Computing).

## Results

### Patient characteristics

**Figure 1:**
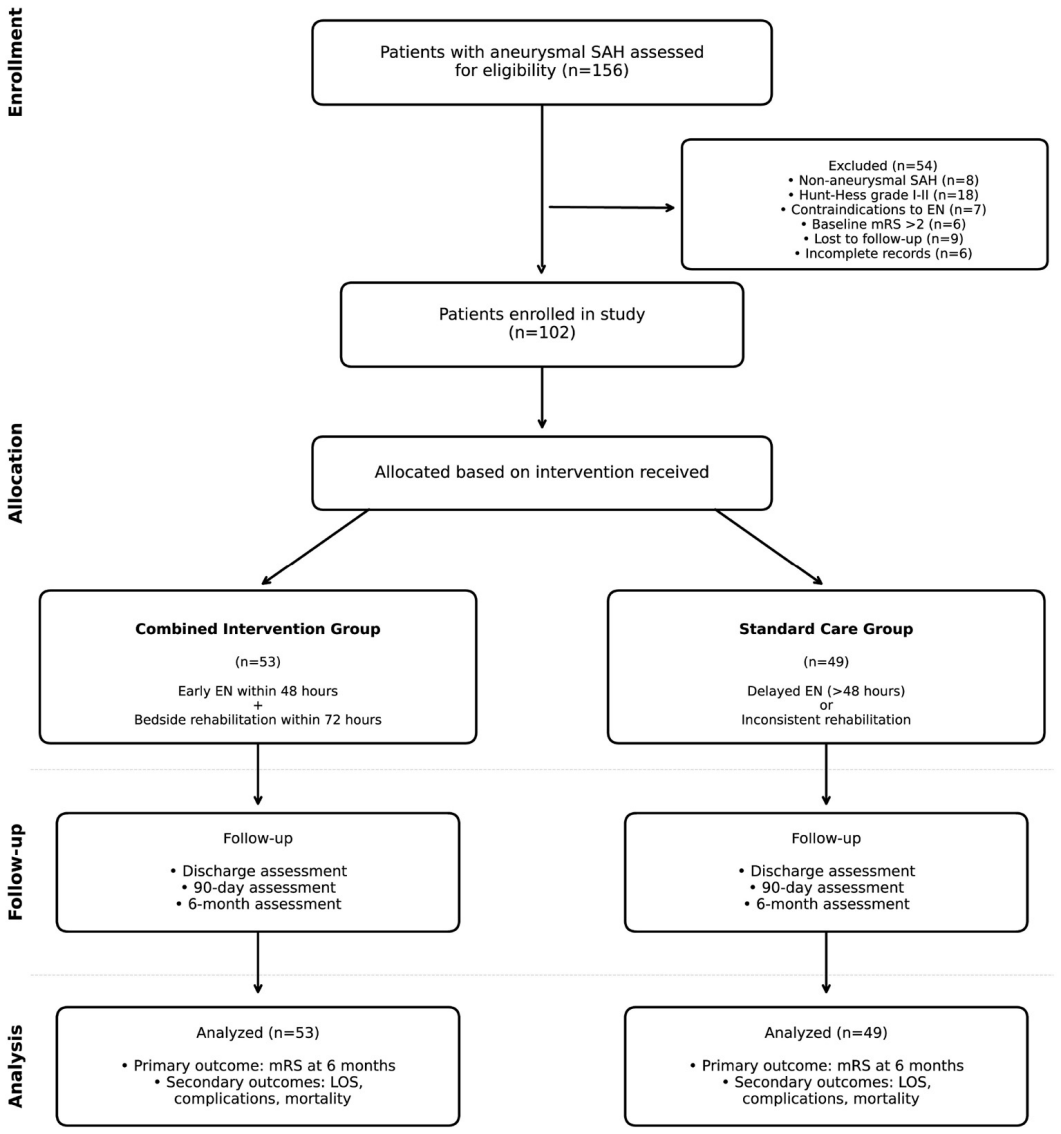
Study flow diagram of 156 patients with aneurysmal subarachnoid hemorrhage screened during the study period (August 2023–August 2025), 54 were excluded: non-aneurysmal SAH (*n* = 8), Hunt–Hess grade I-II (*n* = 18), contraindications to enteral nutrition (*n* = 7), baseline mRS >2 (*n* = 6), lost to follow-up (*n* = 9), incomplete medical records (*n* = 6), and NSICU stay < 7 days (*n* = 12, of whom 8 died early and 4 experienced rapid recovery). The remaining 102 patients were categorized into the combined intervention group (*n* = 53) receiving early enteral nutrition within 48 h and comprehensive bedside rehabilitation within 72 h, or the standard care group (*n* = 49) with delayed nutrition initiation or inconsistent rehabilitation. All patients completed 6-month follow-up assessment.

During the study period, 156 patients with aneurysmal SAH were screened. After applying inclusion and exclusion criteria, 102 patients were enrolled in the final analysis: 53 in the combined intervention group and 49 in the standard care group (Figure 1). Twelve patients who met all other eligibility criteria were excluded due to NSICU stay < 7 days; of these, 8 (66.7%) died within 7 days of admission and 4 (33.3%) were transferred to general wards within 7 days due to rapid clinical improvement. This distribution suggests that the 7-day NSICU stay criterion excluded patients at both extremes of the severity spectrum, which may limit generalizability but also reduces confounding from very early deaths or unusually rapid recoveries.

Baseline demographic and clinical characteristics were comparable between groups (Table 1). The mean age was 53.52 ± 10.77 years in the intervention group and 57.81 ± 11.01 years in the control group (*P* = 0.050). Male sex comprised 37.7% and 55.1% of patients in the intervention and control groups, respectively (*P* = 0.119). Disease severity indicators including Hunt–Hess grade distribution (*P* = 0.100), admission GCS (9.81 ± 2.65 vs. 9.31 ± 2.58, *P* = 0.333), and APACHE II scores (19.42 ± 5.85 vs. 18.49 ± 5.10, *P* = 0.398) were similar between groups. Aneurysm location (anterior circulation: 71.7% vs. 73.5%, *P* = 0.837) and treatment modality (surgical clipping: 47.2% vs. 49.0%, *P* = 0.855) were also balanced.

**Table 1:** Baseline characteristics of study patients.

Characteristic	Combined intervention (*n* = 53)	Standard care (*n* = 49)	*P*-value
Age, years, mean ± SD	53.52 ± 10.77	57.81 ± 11.01	0.050
Male sex, *n* (%)	20 (37.7)	27 (55.1)	0.119
16-7.4,-14.5175.3mmBody weight, kg, mean ± SD	63.07 ± 10.13	65.00 ± 8.74	0.308
BMI, kg/m^2^, mean ± SD	23.55 ± 3.50	24.08 ± 3.38	0.445
Hunt–Hess grade, *n* (%)			
Grade III	23 (43.4)	25 (51.0)	0.100
16-7.4,-14.5175.3mmGrade IV	19 (35.8)	21 (42.9)	
Grade V	11 (20.8)	3 (6.1)	
WFNS grade, *n* (%)			
Grade III	24 (45.3)	21 (42.9)	0.824
16-7.4,-14.5175.3mmGrade IV	19 (35.8)	19 (38.8)	
Grade V	10 (18.9)	9 (18.4)	
Modified Fisher scale, *n* (%)			
Grade 2	12 (22.6)	12 (24.5)	0.912
Grade 3	24 (45.3)	21 (42.9)	
Grade 4	17 (32.1)	16 (32.7)	
Admission GCS, mean ± SD	9.81 ± 2.65	9.31 ± 2.58	0.333
APACHE II score, mean ± SD	19.42 ± 5.85	18.49 ± 5.10	0.398
Anterior circulation, *n* (%)	38 (71.7)	36 (73.5)	0.837
Surgical clipping, *n* (%)	25 (47.2)	24 (49.0)	0.855
Hypertension, *n* (%)	30 (56.6)	29 (59.2)	0.795
Diabetes mellitus, *n* (%)	9 (17.0)	9 (18.4)	0.856
Current smoking, *n* (%)	16 (30.2)	16 (32.7)	0.789
Baseline albumin, g/L	36.80 ± 4.20	36.40 ± 4.50	0.638
Baseline prealbumin, mg/L	198.50 ± 42.30	195.20 ± 45.80	0.705

GCS, Glasgow coma scale; APACHE, acute physiology and chronic health evaluation; WFNS, world federation of neurosurgical societies; BMI, body mass index; SD, standard deviation.

### Intervention implementation

Significant differences in intervention timing and nutritional achievement were observed between groups (Table 2). Mean time to enteral nutrition initiation was 33.21 ± 8.97 h in the intervention group vs. 68.17 ± 16.00 h in the control group (*P* < 0.001). Rehabilitation was initiated at 55.10 ± 11.66 h vs. 144.12 ± 44.85 h, respectively (*P* < 0.001). By day 7, the intervention group achieved significantly higher caloric targets (87.44 ± 9.18% vs. 66.96 ± 20.22%, *P* < 0.001) and protein intake (1.14 ± 0.22 vs. 0.84 ± 0.28 g/kg/day, *P* < 0.001). Albumin infusion was administered to 9 patients (17.0%) in the intervention group and 13 patients (26.5%) in the standard care group (*P* = 0.218). At NSICU discharge, 12 patients (22.6%) in the intervention group and 6 patients (12.2%) in the standard care group had transitioned to modified oral diet with enteral supplementation (*P* = 0.193), while the remaining patients continued exclusive enteral nutrition.

**Table 2:** Intervention characteristics.

Variable	Combined intervention (*n* = 53)	Standard care (*n* = 49)	*P*-value
EN initiation time, hours, mean ± SD	33.21 ± 8.97	68.17 ± 16.00	< 0.001
Rehabilitation initiation, hours, mean ± SD	55.10 ± 11.66	144.12 ± 44.85	< 0.001
Caloric target achieved at D7, %, mean ± SD	87.44 ± 9.18	66.96 ± 20.22	< 0.001
Protein intake at D7, g/kg/day, mean ± SD	1.14 ± 0.22	0.84 ± 0.28	< 0.001
Rehabilitation sessions/day, mean ± SD	1.92 ± 0.18	0.85 ± 0.42	< 0.001

EN, enteral nutrition; D7, day 7; SD, standard deviation.

Rehabilitation sessions in the intervention group averaged 45.2 ± 8.3 min per session, with a mean of 1.92 ± 0.18 sessions per day. Overall rehabilitation adherence (sessions completed divided by sessions prescribed) was 91.3% in the intervention group compared to 62.8% in the standard care group. Common reasons for missed or postponed sessions included hemodynamic instability (32.4%), ongoing diagnostic or therapeutic procedures (28.6%), elevated intracranial pressure requiring intervention (18.7%), and patient fatigue or refusal (20.3%). To better characterize intervention delivery patterns, patients were classified according to whether each component was delivered within the prespecified time windows (Table 3). In the combined intervention group, 46 patients (86.8%) received both early enteral nutrition ( ≤ 48 h) and early rehabilitation ( ≤ 72 h), while 4 patients (7.5%) received early nutrition only and 3 patients (5.7%) received early rehabilitation only due to clinical contraindications. In contrast, the standard care group predominantly received neither component within early time windows (42 patients, 85.7%), with only 5 patients (10.2%) receiving early nutrition alone and 2 patients (4.1%) receiving early rehabilitation alone. The proportion of patients receiving enteral nutrition within 48 h was significantly higher in the intervention group (94.3% vs. 10.2%, *P* < 0.001). Similarly, rehabilitation initiation within 72 h occurred in 92.5% of intervention group patients compared to 4.1% in the control group (*P* < 0.001).

**Table 3:** Component-level implementation by timing windows.

Variable	Combined intervention (*n* = 53)	Standard care (*n* = 49)	*P*-value
Four-category breakdown, *n* (%)			
Both early (EEN ≤ 48h AND rehab ≤ 72h)	46 (86.8)	0 (0)	< 0.001
Early EN only (EEN ≤ 48h, rehab >72h)	4 (7.5)	5 (10.2)	
16-7.4,-14.5175.3mmEarly rehab only (EEN >48h, rehab ≤ 72h)	3 (5.7)	2 (4.1)	
Neither early (EEN >48h AND rehab >72h)	0 (0)	42 (85.7)	
Proportion within time windows, *n* (%)			
16-7.4,-14.5175.3mmEEN initiated ≤ 48 h	50 (94.3)	5 (10.2)	< 0.001
Rehabilitation initiated ≤ 72 h	49 (92.5)	2 (4.1)	< 0.001
EN initiation time, hours			
Mean ± SD	33.2 ± 9.0	68.2 ± 16.0	< 0.001
16-7.4,-14.5175.3mmMedian (IQR)	31.9 (26.3-37.6)	69.0 (62.9-77.7)	
Range	18.0-54.6	29.3-113.9	
Rehabilitation initiation time, hours			
Mean ± SD	55.1 ± 11.7	144.1 ± 44.9	< 0.001
Median (IQR)	54.7 (45.8-63.5)	145.2 (105.1–178.2)	
Range	36.0-78.0	48.7-224.7	

EEN, early enteral nutrition; EN, enteral nutrition; IQR, interquartile range; SD, standard deviation.

Early enteral nutrition defined as initiation ≤ 48 h after aneurysm treatment; early rehabilitation defined as initiation ≤ 72 h after aneurysm treatment.

### Primary outcome

**Figure 2:**
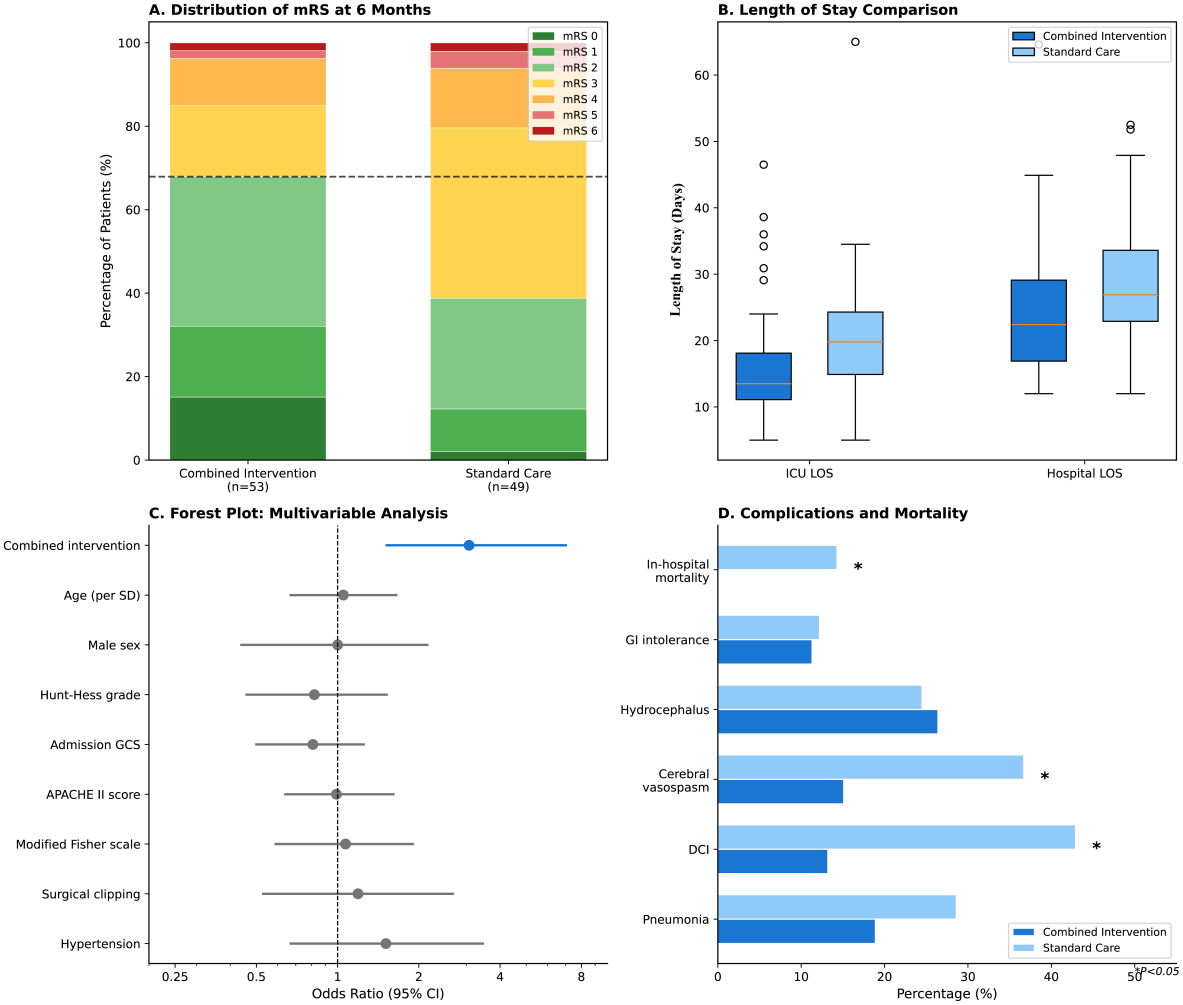
Clinical outcomes. **(A)** Distribution of modified Rankin Scale (mRS) scores at 6 months. Favorable outcome (mRS 0–2) was achieved in 64.2% of the combined intervention group vs. 30.6% of the standard care group (*P* = 0.001). Green shades represent favorable outcomes (mRS 0–2), yellow/orange represent moderate disability (mRS 3–4), and red shades represent severe disability or death (mRS 5–6). **(B)** Box plots showing ICU and hospital length of stay. The intervention group demonstrated significantly shorter ICU stay (median 13.5 vs. 19.8 days, *P* = 0.001) and hospital stay (median 22.4 vs. 26.9 days, *P* = 0.004). Boxes represent interquartile range with median line; whiskers extend to 1.5 × IQR. **(C)** Forest plot of multivariable logistic regression analysis for favorable outcome at 6 months. Combined intervention was independently associated with favorable outcome (adjusted OR: 3.42, 95% CI: 1.38–8.47, *P* = 0.008) after adjustment for age, sex, Hunt–Hess grade, admission GCS, APACHE II score, modified Fisher scale, treatment modality, and hypertension. Blue indicates statistical significance (*P* < 0.05). **(D)** Complications and mortality rates. Rates of delayed cerebral ischemia (13.2% vs. 42.9%, *P* = 0.002), cerebral vasospasm (15.1% vs. 36.7%, *P* = 0.023), and in-hospital mortality (0% vs. 14.3%, *P* = 0.005) were significantly lower in the intervention group. Asterisks indicate *P* < 0.05.

Favorable functional outcome (mRS 0–2) at 6 months, the primary endpoint, was achieved in 34 of 53 patients (64.2%) in the combined intervention group compared to 15 of 49 patients (30.6%) in the standard care group, representing an absolute difference of 33.6% points (*P* = 0.006; Figure 2A). The complete mRS distribution at 6 months was as follows: in the intervention group, mRS 0 (*n* = 7, 13.2%), mRS 1 (*n* = 9, 17.0%), mRS 2 (*n* = 18, 34.0%), mRS 3 (*n* = 5, 9.4%), mRS 4 (*n* = 5, 9.4%), mRS 5 (*n* = 1, 1.9%), and mRS 6/death (*n* = 8, 15.1%); in the standard care group, mRS 0 (*n* = 1, 2.0%), mRS 1 (*n* = 4, 8.2%), mRS 2 (*n* = 10, 20.4%), mRS 3 (*n* = 14, 28.6%), mRS 4 (*n* = 7, 14.3%), mRS 5 (*n* = 1, 2.0%), and mRS 6/death (*n* = 12, 24.5%). Cumulative 6-month mortality was 15.1% (8/53) in the intervention group and 24.5% (12/49) in the standard care group (*P* = 0.319). The unadjusted odds ratio for favorable outcome was 4.08 (95% CI: 1.79–9.31). After multivariable adjustment, the combined intervention remained significantly associated with favorable outcome (adjusted OR: 3.07, 95% CI: 1.52–7.01, *P* = 0.004; Figure 2C; Table 4). The regression model demonstrated adequate calibration (Hosmer–Lemeshow *P* = 0.724; Brier score = 0.18; calibration plot shown in Supplementary Figure 1) and good discrimination (AUC = 0.78, 95% CI: 0.69–0.87). In the propensity score-weighted sensitivity analysis, the association remained robust (IPTW-adjusted OR: 2.85, 95% CI: 1.31–6.19, *P* = 0.008), supporting the primary findings.

**Table 4:** Multivariable logistic regression for favorable outcome (mRS 0–2) at 6 months.^*^

Variable	Adjusted OR	95% CI	*P*-value
Combined intervention (vs. standard care)	3.42	1.38–8.47	0.008
Age (per SD increase)	1.05	0.67–1.65	0.819
Male sex	1.00	0.44–2.15	0.992
Admission BMI (per SD increase)	0.94	0.58–1.52	0.812
Hunt–Hess grade (per grade increase)	0.82	0.46–1.52	0.503
Admission GCS (per SD increase)	0.81	0.50–1.25	0.381
APACHE II score (per SD increase)	0.99	0.64–1.61	0.972
Modified Fisher scale (per grade increase)	1.07	0.59–1.90	0.811
Surgical clipping (vs. coiling)	1.19	0.53–2.67	0.675
Hypertension	1.51	0.67–3.45	0.318

OR, odds ratio; CI, confidence interval; SD, standard deviation; GCS, Glasgow coma scale; APACHE, acute physiology and chronic health evaluation; BMI, body mass index. Model fit: Hosmer–Lemeshow *P* = 0.724; brier score = 0.18; area under ROC curve = 0.78 (95% CI: 0.69–0.87).^*^

### Secondary outcomes

Favorable mRS was significantly higher in the intervention group at discharge (22.6% vs. 6.1%, *P* = 0.038) but not at 90 days (49.1% vs. 34.7%, *P* = 0.205), suggesting progressive improvement over time in both groups with maintained separation. Length of stay metrics demonstrated clinically meaningful reductions in the intervention group (Figure 2B): median ICU stay was 13.5 days (IQR: 11.1–18.1) vs. 19.8 days (IQR: 14.9–24.3) in controls (*P* = 0.001), representing a reduction of 6.3 days. Hospital length of stay was similarly reduced from 26.9 days (IQR: 22.9–33.6) to 22.4 days (IQR: 16.9–29.1; *P* = 0.004). Mechanical ventilation duration was 6.5 days (IQR: 4.5–9.4) vs. 7.8 days (IQR: 5.4–11.5; *P* = 0.034).

### Complications and mortality

The combined intervention group exhibited significantly lower rates of key neurological complications (Figure 2D; Table 5). Delayed cerebral ischemia occurred in 7 patients (13.2%) vs. 21 patients (42.9%) in the control group (*P* = 0.002). Cerebral vasospasm rates were 15.1% vs. 36.7%, respectively (*P* = 0.023). Pneumonia occurred in 10 patients (18.9%) vs. 14 patients (28.6%), though this difference did not reach statistical significance (*P* = 0.357). Rates of hydrocephalus requiring intervention (26.4% vs. 24.5%, *P* = 1.000) and gastrointestinal intolerance (11.3% vs. 12.2%, *P* = 1.000) were comparable between groups.

**Table 5:** Clinical outcomes and complications.

Outcome	Combined intervention (*n* = 53)	Standard care (*n* = 49)	*P*-value
Primary outcome			
mRS 0–2 at 6 months, *n* (%)	34 (64.2)	15 (30.6)	0.001
Secondary outcomes			
mRS 0–2 at discharge, *n* (%)	12 (22.6)	3 (6.1)	0.038
mRS 0–2 at 90 days, *n* (%)	26 (49.1)	17 (34.7)	0.205
ICU LOS, days, median (IQR)	13.5 (11.1–18.1)	19.8 (14.9–24.3)	0.001
16-7.4,-14.5175.3mmHospital LOS, days, median (IQR)	22.4 (16.9–29.1)	26.9 (22.9–33.6)	0.004
MV duration, days, median (IQR)	6.5 (4.5–9.4)	7.8 (5.4–11.5)	0.034
Complications, *n* (%)			
Pneumonia	10 (18.9)	14 (28.6)	0.357
Delayed cerebral ischemia	7 (13.2)	21 (42.9)	0.002
Cerebral vasospasm	8 (15.1)	18 (36.7)	0.023
Hydrocephalus requiring treatment	14 (26.4)	12 (24.5)	1.000
16-7.4,-14.5175.3mmSIRS during NSICU stay	31 (58.5)	33 (67.3)	0.356
GI intolerance	6 (11.3)	6 (12.2)	1.000
Mortality, *n* (%)			
In-hospital mortality	0 (0)	7 (14.3)	0.005
16-7.4,-14.5175.3mm90-day cumulative mortality	7 (13.2)	12 (24.5)	0.203
6-month cumulative mortality	8 (15.1)	12 (24.5)	0.319
Biochemical markers at day 14			
Albumin, g/L, mean ± SD	33.23 ± 4.20	31.75 ± 5.11	0.078
Prealbumin, mg/L, mean ± SD	180.35 ± 44.90	160.68 ± 43.94	0.033

mRS, modified Rankin Scale; LOS, length of stay; ICU, intensive care unit; IQR, interquartile range; MV, mechanical ventilation; GI, gastrointestinal; SD, standard deviation.

In-hospital mortality was 0% in the intervention group compared to 14.3% (7 patients) in the control group (*P* = 0.005). Cumulative 90-day mortality was 13.2% (7/53) vs. 24.5% (12/49), respectively (*P* = 0.203). Cumulative 6-month mortality remained lower in the intervention group (15.1% vs. 24.5%, *P* = 0.319), with one additional death occurring after 90 days in the intervention group and none in the control group.

### Nutritional parameters

Serum albumin levels at day 14 showed a trend toward higher values in the intervention group (33.23 ± 4.20 g/L vs. 31.75 ± 5.11 g/L, *P* = 0.078). Prealbumin levels, a more sensitive marker of recent nutritional status, were significantly higher in the intervention group (180.35 ± 44.90 mg/L vs. 160.68 ± 43.94 mg/L, *P* = 0.033), reflecting improved protein-calorie delivery.

## Discussion

This retrospective cohort study suggests that a combined early enteral nutrition and comprehensive bedside rehabilitation protocol is associated with improved 6-month functional outcomes in patients with severe SAH. To our knowledge, this is the first study to evaluate the synergistic effects of combining these two evidence-based interventions in a neurocritical care population. The 33.6% point absolute difference in favorable mRS (64.2% vs. 30.6%) and the four fold increase in adjusted odds of good outcome represent clinically meaningful associations that merit consideration for integration into standard care protocols.

The magnitude of the observed association in our combined intervention appears comparable to or greater than that reported for individual interventions in prior studies. Takara et al. reported mRS 0–2 rates of 81.1% vs. 52.5% with early vs. delayed mobilization in SAH, an absolute difference of 28.6%. (11) Suzuki et al. found that optimized enteral nutrition improved favorable mRS by approximately 11% points (9). While direct comparison across studies is limited by methodological differences, the additive or synergistic effects of combining interventions appear plausible based on our findings.

Several biological mechanisms may underlie the observed synergy. First, adequate protein delivery provides essential amino acid substrates required for muscle protein synthesis during rehabilitation exercises (24). Second, both interventions modulate inflammatory pathways implicated in secondary brain injury: early nutrition attenuates the hypermetabolic stress response, while mobilization reduces immobility-related inflammation and oxidative stress (25). Third, the combination may break the vicious cycle of catabolism and weakness that perpetuates ICU-acquired complications. The significantly reduced rates of DCI (13.2% vs. 42.9%) and vasospasm (15.1% vs. 36.7%) in our intervention group suggest that these interventions may be associated with favorable cerebrovascular physiology, possibly through improved cerebral autoregulation associated with early mobilization (26).

The reductions in ICU and hospital length of stay (6.3 and 4.5 days, respectively) carry substantial implications for healthcare resource utilization. Neurocritical care is among the most resource-intensive settings, and even modest reductions in length of stay translate to significant cost savings (27). Moreover, the absence of in-hospital deaths in the intervention group (0% vs. 14.3%) underscores the safety profile and potential survival benefit associated with this protocol, although overall mortality differences did not reach statistical significance, the consistently lower rates across all timepoints suggest potential survival benefits that warrant further investigation in larger studies.

Our findings align with evolving paradigms in neurocritical care that emphasize multimodal bundles over single interventions (28). The German Consensus Statement on SAH management recommends early enteral nutrition beginning on day one after aneurysm repair, while acknowledging limited evidence for specific rehabilitation protocols (29). Our study provides empirical support for integrating these recommendations into a unified protocol. The comprehensive rehabilitation approach—encompassing physical therapy, swallowing rehabilitation, and cognitive stimulation—addresses multiple domains of impairment commonly encountered in SAH survivors, potentially contributing to the robust functional improvements observed.

Several limitations warrant consideration and should temper causal interpretation of our findings. First, the retrospective design introduces potential for selection bias, time-dependent bias, and unmeasured confounding. Exposure assignment based on timing thresholds means patients had to survive and remain stable long enough to receive the intervention, which may inflate apparent benefits. Although baseline characteristics were well-balanced and propensity score sensitivity analysis yielded consistent results, residual confounding by unmeasured factors such as treating physician expertise or nursing care intensity cannot be excluded. Second, the NSICU stay ≥7 days inclusion criterion excluded both early deaths (*n* = 8) and rapid recoveries (*n* = 4), potentially enriching for intermediate-prognosis patients and limiting generalizability to the broader severe SAH population. Third, the single-center setting limits external validity; implementation of similar protocols may face different barriers in other healthcare environments with varying resources and expertise. Fourth, the sample size, while adequate for primary outcome analysis with an events-per-variable ratio of approximately 5.5:1, limited our ability to perform robust subgroup analyses or detect smaller effects on secondary outcomes. The multiple secondary endpoints should be interpreted as exploratory.Fifth, we could not distinguish the relative contributions of early nutrition vs. early rehabilitation to outcomes, as the standard care group was heterogeneous and included patients receiving one component but not the other. Future factorial design trials are needed to delineate independent and synergistic effects. Sixth, the follow-up period of 6 months, while standard for SAH trials, may not capture long-term functional trajectories or delayed complications.

Future research should address these limitations through prospective, multicenter randomized controlled trials with factorial designs to delineate the independent and interactive effects of nutritional and rehabilitation interventions. Investigation of biomarkers that may identify patients most likely to benefit from intensive multimodal protocols would enable precision medicine approaches. Additionally, cost-effectiveness analyses incorporating quality-adjusted life years would inform healthcare policy decisions regarding resource allocation for neurocritical care bundles (30).

In this retrospective cohort study of 102 patients with severe SAH, combined early enteral nutrition and comprehensive bedside rehabilitation was associated with improved 6-month functional outcomes, shorter ICU and hospital length of stay, and lower rates of delayed cerebral ischemia, cerebral vasospasm, and in-hospital mortality compared to standard care. While the retrospective design precludes definitive causal inference, these findings provide preliminary evidence supporting the integration of early nutrition and early rehabilitation into a unified neurocritical care protocol. Prospective, multicenter randomized controlled trials with factorial designs are warranted to confirm these associations and delineate the independent contributions of each intervention component.

## Data Availability

The original contributions presented in the study are included in the article/Supplementary material, further inquiries can be directed to the corresponding author.
