# Supplementary material for: Impact of early enteral nutrition combined with bedside rehabilitation on functional outcomes and length of stay in patients with severe subarachnoid hemorrhage: a retrospective cohort study
Source: Front Neurol. 2026 Apr 8;17:1768799. doi: 10.3389/fneur.2026.1768799 (PMC13099312; doi:10.3389/fneur.2026.1768799)
Supplement: Supplementary file 1 [file Image_1.pdf]

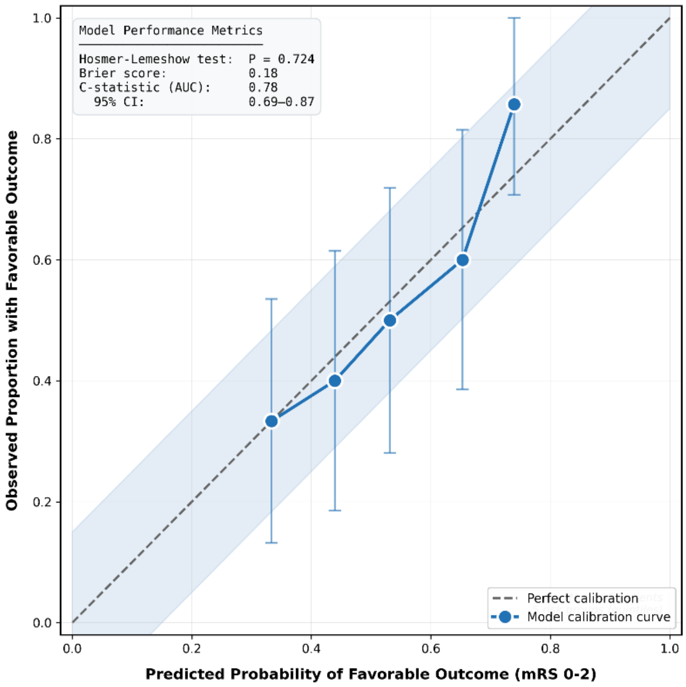

Supplementary Figure 1. Calibration plot for the multivariable logistic regression model predicting favorable functional outcome (mRS 0-2) at 6 months.

The calibration plot displays the agreement between model-predicted probabilities (x-axis) and observed proportions of favorable outcome (y-axis). Patients were divided into five risk groups based on predicted probability quintiles. Blue circles represent the mean predicted probability and observed proportion for each group, connected by solid lines; error bars indicate 95% confidence intervals based on binomial standard errors. The dashed diagonal line indicates perfect calibration, where predicted probabilities exactly match observed outcomes. The shaded band represents an approximate 95% confidence region around perfect calibration. Close alignment of the calibration curve with the diagonal reference line indicates adequate model calibration. Model performance metrics are displayed in the inset: the Hosmer-Lemeshow goodness-of-fit test (P = 0.724) indicates no significant lack of fit; the Brier score of 0.18 suggests good overall calibration; and the area under the receiver operating characteristic curve (C-statistic) of 0.78 (95% CI: 0.69–0.87) demonstrates acceptable discrimination. mRS, modified Rankin Scale; CI, confidence interval; AUC, area under the curve.
